# Genomic Characterization of Strains From a Cluster of Infant Botulism Type A in a Small Town in Colorado, United States

**DOI:** 10.3389/fmicb.2021.688240

**Published:** 2021-07-13

**Authors:** Lori Gladney, Jessica L. Halpin, Carolina Lúquez

**Affiliations:** National Botulism Laboratory, Enteric Diseases Laboratory Branch, Division of Foodborne, Waterborne and Environmental Diseases, National Center for Emerging Zoonotic and Infectious Diseases, Centers for Disease Control and Prevention, Atlanta, GA, United States

**Keywords:** *Clostridium botulinum*, botulism, infant botulism cluster, single-nucleotide polymorphism, high-quality SNP typing

## Abstract

Three cases of infant botulism were reported in a small Colorado town between 1981 and 1984. The first two cases occurred in 1981, 6 months apart, and the third case occurred in 1984. *Clostridium botulinum* type A was isolated from stool of all three case patients and from environmental samples of the patient’s homes. An epidemiological investigation and follow-up study were conducted from 1981 to 1986 and concluded the cases were likely related. In this study, we sought to determine whether the *C. botulinum* type A clinical isolates were related to each other and to isolates obtained from environmental samples. We performed whole genome sequencing (WGS) for 17 isolates associated with this potential cluster of infant botulism. Fifteen isolates were confirmed to be *C. botulinum* type A(B) and contained botulinum toxin gene subtypes A1 and B5 by WGS; these strains formed a monophyletic cluster in a phylogeny and were considered closely related to each other (0–18 high-quality single-nucleotide polymorphisms), but distinct from other *C. botulinum* type A(B) in Colorado and elsewhere in the United States. Results of our study suggest that the three infant botulism cases could have represented a cluster due to a *C. botulinum* type A(B) strain present in the environment.

## Introduction

*Clostridium botulinum* are spore-forming Gram-positive bacteria that produce a potent neurotoxin responsible for the severe paralytic disease botulism. In the United States, there are on average 153 cases of botulism each year, and the majority (110 cases per year) are attributed to infant botulism.^1^ *C. botulinum* strains produce seven well-characterized botulinum toxin types (A-G), although human illness in the United States is primarily caused by toxin types A and B, followed by E and F. Some *C. botulinum* strains, denominated bivalent, can produce more than one toxin type (Sobel, 2005; Raphael et al., 2014). *C. botulinum* is a diverse species and has historically been characterized into four metabolic groups designated Groups I, II, III, and IV, based on biochemical and microbiological traits (Raphael et al., 2014; Smith et al., 2015; Williamson et al., 2016). Group I consists of proteolytic strains that produce botulinum toxin types A, B, and F (Smith et al., 2015; Williamson et al., 2016). Group I strains are most often associated with human clinical cases and are the most common cause of botulism cases in the United States. Group I also includes bivalent strains (Ab, Af, Ba, and Bf), and strains designated type A(B), which carry types A and B botulinum neurotoxin (*bont*) genes, but only produce type A toxin (Franciosa et al., 1994; Hill et al., 2007). These *C. botulinum* A(B) strains have been discovered to be common among the US botulism cases (Raphael et al., 2014; Halpin et al., 2017).

The disease botulism can be classified into four natural-occurring forms: foodborne botulism (through ingestion of foods contaminated with botulinum toxin), wound botulism (through spores that germinate leading to growth in wounds, and toxin production *in situ*), infant botulism (through intestinal colonization in infants less than 1 year old), and adult intestinal colonization (through intestinal colonization of patients older than 1 year; Sobel, 2005). Infant botulism is the most common type of botulism in the United States, and it occurs in persons under 1 year old, through ingestion of *C. botulinum* spores that germinate in the intestines, leading to growth and production of botulinum toxin (Sobel, 2005). Spores of *C. botulinum* are commonly found in soil, and therefore, it has been proposed as the most probable source of spores in infant botulism cases (Koepke et al., 2008).

Here, we present a follow-up study, to characterize *C. botulinum* strains isolated from clinical and environmental samples associated with the investigation conducted by Istre et al. (1986), these cases were of interest since transmission of botulism is not considered communicable (does transmit from person to person) and the incidence of infant botulism is extremely low. The first two cases occurred in 1981, 6 months apart, while the third case occurred in September of 1984. All three infants lived in the same neighborhood, within 800 m of each other. The three cases were confirmed by detection of botulinum neurotoxin type A in patient stools using the mouse bioassay (Istre et al., 1986). *C. botulinum* type A was isolated from all three of the patient’s stools. *C. botulinum* type A was also present in cultures of environmental samples taken in 1982 and 1985, following the second and third cases. Specifically, *C. botulinum* type A was found in soil and vacuum dust samples taken from the homes of the second and third cases and also from the crib of the second case patient (Istre et al., 1986). *C. botulinum* type A was also found in soil of the first case patient’s home after the fact, but no house dust samples were obtained as the family moved prior to the onset of the second case (Istre et al., 1986). This investigation prompted a study in 1986, conducted by CDC, to test additional environmental samples from homes of infants in the community who were considered healthy control infants, as they were presumed not to be experiencing symptoms of botulism. Stool samples from these healthy infants were also collected. Ten stool samples, 22 soil samples, and 13 vacuum dust samples were collected: 91% (20/22) of soil samples from the homes of control infants yielded *C. botulinum* type A, and 77% (10/13) of vacuum dust samples from the homes of control infants also yielded *C. botulinum* type A. *C. botulinum* type A was not identified in the stool of any control infants; however, *C. botulinum* type B was found in the stools of two control infants (unpublished data). In this study, we employed a whole genome sequencing (WGS) approach to further characterize the clinical and environmental isolates associated with this investigation and to determine (1) whether the three cases represented a cluster of infant botulism cases and (2) whether the *C. botulinum* type A clinical isolates were related to the isolates found in environmental samples.

## Materials and Methods

### Strains

Seventeen *C. botulinum* isolates were selected from the CDC strain collection for further characterization (refer to Table 1): four type A isolates from patient stool samples associated with cases one and three (unfortunately, the isolate from case 2 was not available in CDC’s strain collection), two type B isolates from stools of two healthy infants, and 11 type A isolates from environmental samples (from cases two and three and the homes of six healthy infants). Eight of the isolates were from the original investigation by Istre et al., 1986, and nine isolates were from the unpublished study in 1986. Additional isolates for geographical perspective were also included in the study, based on availability of WGS data (refer to Supplementary Table 1). Strains were grown at 35°C under anaerobic conditions in Trypticase Peptone Yeast Extract (Remel, Lenexa, KS). All strains were coded prior to the study to comply with a human subjects research protocol. DNA was extracted using a modified MasterPure DNA extraction protocol (Epicenter, Madison, WI; Halpin et al., 2019).

**Table 1:** *C. botulinum* strains included in this study.

CDC strain No./NCBI accession	Year	Botulism type	Source type	Serotype *mouse bioassay*^c^	Toxin subtype *WGS*	Case information
CDC36910 SAMN17597327	1981	Infant	Stool	A	A1,B5	Case 1 clinical sample
CDC36911 SAMN17597328	1981	Infant	Stool	A	A1,B5	Case 1 clinical sample
CDC37208 SAMN17597330	1982	N/A	Vacuum dust^a^	A	A1,B5	Case 2 environmental sample
CDC37220 SAMN17597329	1982	N/A	Crib springs	A	A1,B5	Case 2 environmental sample
CDC37369 SAMN17597331	1982	N/A	Soil	A	A1,B5	Case 2 environmental sample
CDC37370 SAMN17597332	1982	N/A	Soil	A	A1,B5	Case 2 environmental sample
CDC39239 SAMN17597333	1984	Infant	Stool	A	A1,B5	Case 3 clinical sample
CDC39242 SAMN17597334	1984	Infant	Stool	A	A1,B5	Case 3 clinical sample
CDC31846 SAMN17597318	1986	N/A	Stool	B	B1	Healthy infant 1
CDC31956 SAMN17597320	1986	N/A	Soil	A	A1,B5	Associated with healthy infant 1
CDC31965 SAMN17597321	1986	N/A	Vacuum dust	A	A1,B5	Associated with healthy infant 2
CDC31966 SAMN17597322	1986	N/A	Soil	A	A1,B5	Associated with healthy infant 3
CDC31969 SAMN17597323	1986	N/A	Soil	A	A1,B5	Associated with healthy infant 4
CDC31973 SAMN17597324	1986	N/A	Vacuum dust	A	A1,B5	Associated with healthy infant 4
CDC31851 SAMN17597319	1986	N/A	Stool	B	Non-toxigenic^b^	Healthy infant 5
CDC31976 SAMN17597326	1986	N/A	Vacuum dust	A	A1,B5	Associated with healthy infant 5
CDC31974 SAMN17597325	1986	N/A	Vacuum dust	A	A1,B5	Associated with healthy infant 6

a Vacuum cleaner dust was collected from the grandmother’s home.

b Isolate does not harbor a bont/B gene – strain may have lost a plasmid with the toxin gene.

c Data obtained from previous laboratory results in the 1980s.

### Sequencing

Isolates were sequenced on either the Ion Torrent S5 or Illumina MiniSeq. DNA libraries were prepared for WGS using the Nextera DNA Flex kit (Illumina, San Diego, CA) and sequenced on the MiniSeq (Illumina, San Diego, CA) with 2 × 150 bp chemistry. DNA libraries were also prepared using the Ion Torrent Chef (Thermo Fisher Scientific, Waltham, MA) and sequenced on the Ion Torrent S5 (Thermo Fisher Scientific, Waltham, MA) using previously established methods (Halpin et al., 2019).

### Genome Assembly and Quality Filtering

Genome sequences were downloaded from NCBI or assembled from raw reads using SPAdes. SPAdes version 3.14.0 and the option --careful were used for Illumina reads, while SPAdes version 3.13.0 and the options -sc, --iontorrent, and --careful were used for Ion Torrent reads (Bankevich et al., 2012). Short contigs (<500 bp) were removed using CG-Pipeline script run_assembly_filterContigs.pl (Kislyuk et al., 2010). Sequence accession numbers are associated with NCBI Bioproject PRJNA428620; these can be found in Table 1.

### Toxin Gene Location and Type Determination

*bont* gene subtypes were determined with CLC Genomics Workbench v10 Map to Reference feature (CLC bio, Aarhus, Denmark). Location of *bont* genes was determined using a command-line nucleotide blast with Blast+ version 2.9.0 (Camacho et al., 2009), and their location on either the chromosome or a plasmid was predicted using nucleotide blast of the *C. botulinum* database on NCBI. Seven-gene Multi-Locus Sequence Typing (MLST) types were determined by querying the *C. botulinum* PubMLST database^3^ (Jacobson et al., 2008).

### WGS Analysis

A single-nucleotide polymorphism (SNP) analysis was performed using only the highest quality SNPs. An appropriate reference genome for a high-quality SNP analysis was identified by first querying our in-house genome sequence repository. These in-house sequences were then compared to additional genome sequences on NCBI, using Mashtree (Katz et al., 2019). An appropriate reference was selected based on two criteria: closeness to the study strains in the Mashtree and completeness of the reference assembly. Plasmid sequences were identified in assemblies using PLSDB (Galata et al., 2019) and removed from the reference sequence to avoid erroneous SNP counts due to plasticity of some strains. Read pairs were preprocessed with Lyve-SET version 1.1.4f scripts prior to performing SNP analyses; read pairs were first interleaved with shuffleSplitReads.pl. and then cleaned with run_assembly_trimClean.pl. with the options --min_avg_quality 24 and --nosingletons (Katz et al., 2017). Lyve-SET version 1.1.4f was used for the SNP analysis with the following settings and the cleaned reads: --allowedFlanking 5 --min_alt_frac 0.75 --min_coverage 10 --mask-phages --mask-cliffs --mapper smalt --numcpus 10 --numnodes 50 (Katz et al., 2017). Lyve-SET quality filters for SNP coverage, percent consensus, and flanking distance ensure that only the highest quality SNPs are used in the analysis and that low-quality regions susceptible to recombination and horizontal exchange are not included (Katz et al., 2017). External reference genome sequences were also placed in the Lyve-SET asm folder for WGS read simulation as needed to root the tree. Phylogenetic trees were visualized using Mega7 (Kumar et al., 2016), and SNP distance matrices were visualized using a text editor or Microsoft Excel (Microsoft, Albuquerque, NM).

## Results

### Toxin Gene Location and Type Determination

All 15 *C. botulinum* type A clinical and environmental isolates were confirmed to be A(B), *bont* gene subtypes A1 and B5 by WGS, and MLST type ST6. The *bont* genes were predicted to be on the chromosome for all *C. botulinum* type A(B) isolates associated with this investigation. Interestingly, the type A and type B toxin genes were located on a single 311,258 bp contig in one of the strains (CDC31973). The presence of both type A and type B toxin genes on the chromosome has been seldomly reported in *C. botulinum* type A(B) strains (Franciosa et al., 2009; Gonzalez-Escalona and Sharma, 2020). One *C. botulinum* type B isolate (CDC31846) from healthy infant stool was confirmed to harbor *bont*/B1 gene and was predicted to be on a plasmid. CDC31851, originally classified as *C. botulinum* type B by mouse bioassay in 1986, did not carry a *bont* gene and was determined as non-toxigenic in this study. The absence of the toxin gene in this isolate CDC31851 could have been due to loss of a plasmid. Both CDC31846 and CDC31851 were confirmed to be MLST type ST96.

### WGS Analysis

**Figure 1:**
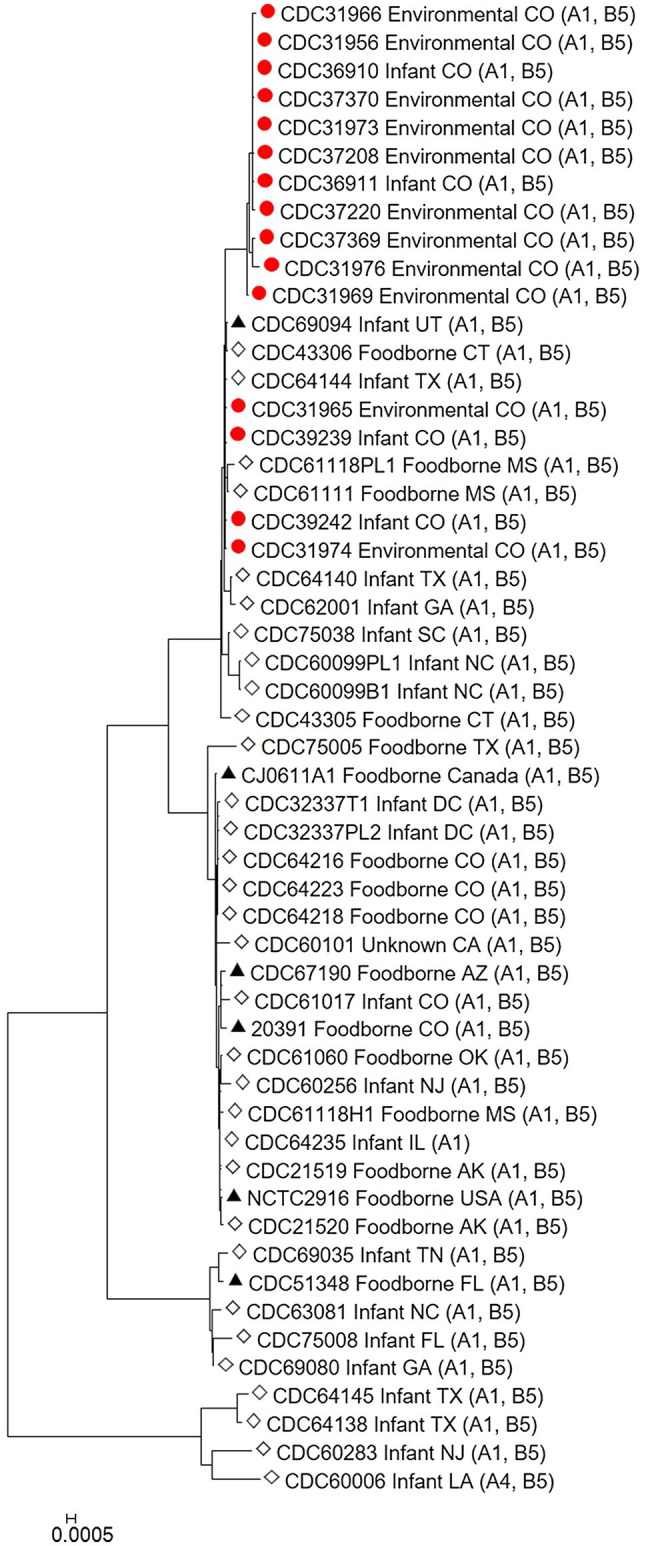
Neighbor-joining tree of *Clostridium botulinum* type A(B) isolates. Tree was drawn using Mashtree and includes study strains harboring both *bont*/A1 and *bont*/B5 genes as described in Table 1 (red circles), reference sequences from NCBI (black triangles), and other unpublished sequences from the CDC reference collection (white diamonds), as described in Supplementary Table 1. Scale bar represents the Mash distance.

The *C. botulinum* type A(B) study strains in Table 1 clustered together in the Mashtree and also with other unrelated *C. botulinum* type A(B) from the United States (Figure 1; Supplementary Table 1). However, they did not cluster with *C. botulinum* type A(B) isolated from other infant and foodborne botulism cases in Colorado (CDC61017, CDC64216, CDC64218, and CDC64223; Figure 1; Supplementary Table 1).

**Figure 2:**
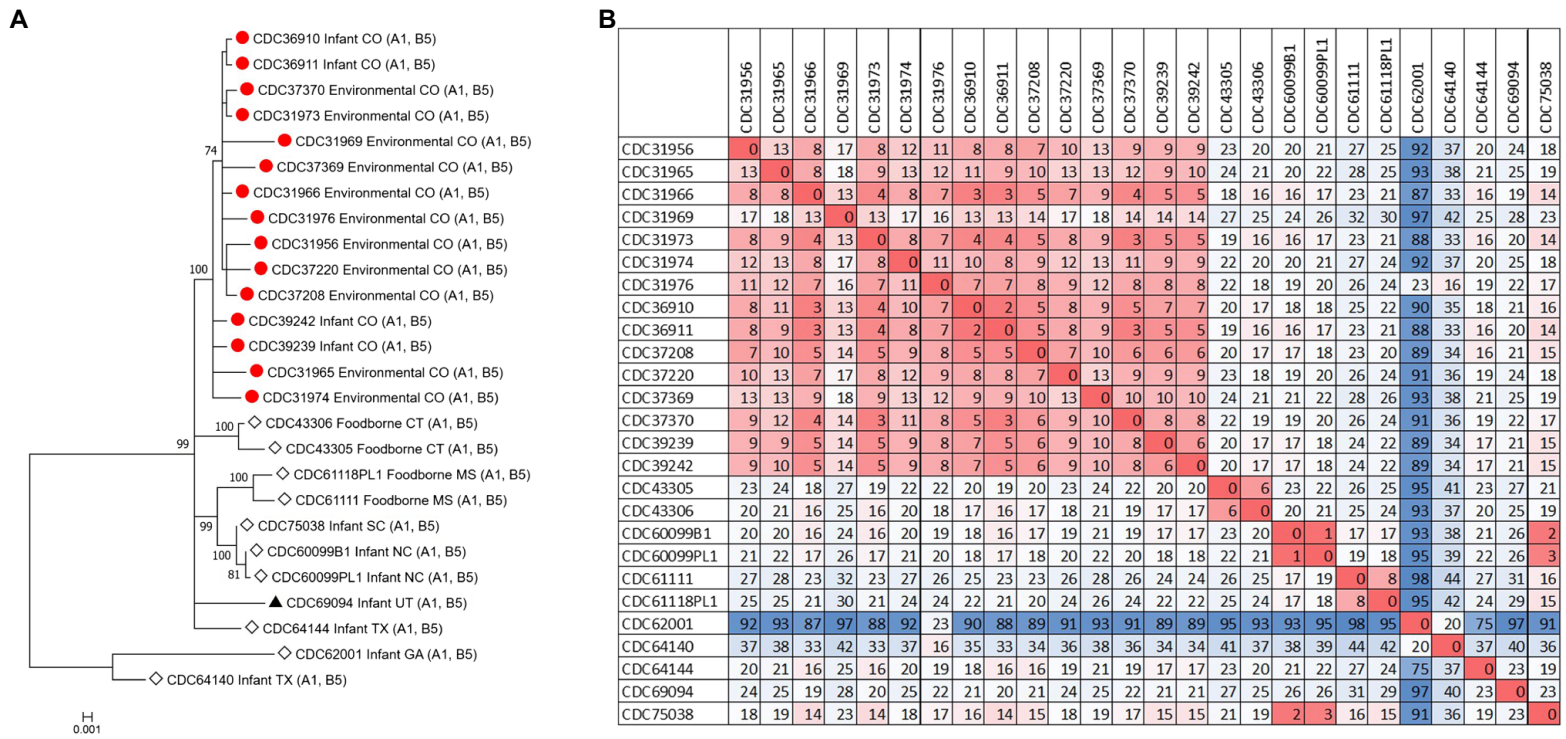
**(A)** Maximum-likelihood whole genome high-quality SNP tree of *C. botulinum* type A(B) isolates harboring both *bont*/A1 and *bont*/B5 genes. Tree was drawn using Lyve-SET and includes study strains as described in Table 1 (red circles), reference sequences from NCBI (black triangles), and other unpublished sequences from *C. botulinum* type A(B) strains from the CDC reference collection (white diamonds). Scale bar represents the number of changes per 100 sites. Bootstrap values greater than 70 are shown. **(B)** Distance matrix of SNP counts from Lyve-SET. The matrix was drawn in Excel using conditional formatting.

The *C. botulinum* type A(B) study strains formed a monophyletic cluster in a Lyve-SET phylogeny and are considered closely related to each other (2–18 high-quality SNPs), but distinct from other closely related *C. botulinum* type A(B) from the United States (CDC43305, CDC43306, CDC69094, and CDC64144; Figures 2A,B). Interestingly, the clinical isolates (CDC36910, CDC36911, CDC39239, and CDC39242) had fewer SNP differences between isolates (5–7 high-quality SNPs), highlighting that the additional diversity (up to 18 SNP differences) in the *C. botulinum* type A(B) cluster is due to the environmental isolates, which were taken at different time points (see Table 1; Figures 2A,B). The two isolates recovered from healthy infant stools (*C. botulinum* type B CDC31846 and the non-toxigenic isolate CDC31851) were 28 SNPs different from each other and were considered unrelated (data not shown).

## Discussion

In this study, we sought to characterize *C. botulinum* type A isolated from clinical specimens from infant botulism cases reported in Colorado between 1981 and 1984 (Istre et al., 1986), and other C. *botulinum* type A isolated from environmental samples. Unfortunately, the clinical isolate associated with the second case was not available in CDC’s strain collection and could not be included as part of this study; while this is a limitation of the study, we did include environmental isolates associated with the second case as part of this study. In a subsequent study done by CDC in 1986, stool samples were collected from 10 healthy control infants as well as soil and dust samples from their homes. The control stools were negative for *C. botulinum* type A, but two infants had *C. botulinum* type B in their stool (unpublished data). It is unclear how the two presumed healthy infants had *C. botulinum* type B in their stool, and to our knowledge, there are no reports of healthy infants harboring *C. botulinum* (Dowell et al., 1977). Additionally, *C. botulinum* type A was found in the environmental samples collected from homes of the healthy control infants. Our findings confirm this and add more to the investigation – the study strains also had a silent *bont*/B5 gene (Franciosa et al., 1994).

The source of *C. botulinum* spores in infant botulism cases is rarely determined, but exposure is thought to be through the environment, by ingestion of microscopic dust particles, or by consumption of honey (Dabritz et al., 2014). Furthermore, our results suggest that soil or dust may have been the probable source of spores in these three infant botulism cases, since the *C. botulinum* type A(B) strains isolated from environmental samples were closely related to the *C. botulinum* type A(B) strains isolated from stool samples. Some diversity (up to 18 SNPs) was observed among the environmental isolates, while very little diversity was observed in the clinical isolates (5 to 7 SNPs). This finding is interesting because the number of SNP differences is similar to what we would expect to see between unrelated clusters of *C. botulinum* A(B) in point source outbreaks (e.g., foodborne outbreaks). Raphael et al. reported SNP ranges of 3–8 between epidemiologically linked *C. botulinum* A(B) isolates of clinical and food pairs in six foodborne outbreaks using a reference-free SNP analysis (Raphael et al., 2014). Halpin et al. reported a range of 2–6 SNPs among *C. botulinum* clinical isolates and an epidemiologically linked contaminated powder infant formula isolate harboring the *bont*/B7 gene using a reference approach in Lyve-SET (Halpin et al., 2019). The differences observed among the environmental isolates in this study may be a result of the diversity of *C. botulinum* spores in the environment where they are collected.

Our study demonstrates the utility of WGS analysis and its enhanced resolution for resolving clusters of *C. botulinum*, in particular of *C. botulinum* type A(B) strains, which have been historically challenging to differentiate (Hill et al., 2007; Raphael et al., 2014; Smith et al., 2015; Halpin et al., 2017). By using Lyve-SET high-quality SNP analysis, we were able to differentiate the study isolates from other closely related *C. botulinum* type A(B) isolates from Colorado and the United States that were not a part of this cluster, but otherwise appear related in a simple distance-based Mashtree; for example, CDC69094, CDC43306, CDC64144, CDC61118PL1, and CDC61111 which cluster in the Mashtree with study strains were unrelated to the study strains in the Lyve-SET phylogeny (see Figures 1, 2A). Katz et al. previously showed the usefulness of Lyve-SET for investigating closely related strains within outbreaks of *Listeria monocytogenes*, *Escherichia coli*, *Salmonella enterica*, and *Campylobacter jejuni* (Katz et al., 2017). At the time of the original investigation, the mouse bioassay was used for confirming the infant botulism cases, and for identifying the botulinum toxin serotype. The mouse bioassay does not, and it is not intended to provide resolution to resolve clusters of closely related strains nor identify an unexpressed botulinum toxin gene. Additional subtyping methods, such as pulsed-field gel electrophoresis, seven-gene MLST, multi-loci variable number of tandem repeat analysis, and amplified fragment length polymorphism analysis, have been utilized to subtype *C. botulinum* isolates in the last 25 years, but none of these subtyping tools can provide the level of resolution needed for resolving clusters of *C. botulinum* type A(B) strains (Raphael et al., 2014; Smith et al., 2015; Halpin et al., 2017). For instance, our study showed that all of the type A(B) isolates shared seven-gene MLST type ST6 with other type A(B) isolates that were not part of the cluster of infant botulism cases (data not shown). Our study builds on previous work to show the utility of reference-based whole genome SNP typing to successfully resolve clusters of *C. botulinum* (Gonzalez-Escalona et al., 2014; Raphael et al., 2014; Williamson et al., 2016; Gonzalez-Escalona and Sharma, 2020). In conclusion, results of this study suggest that these *C. botulinum* type A(B) isolates obtained from infant botulism cases are genetically related to each other and to the environmental isolates obtained from soil and dust samples.

## Data Availability Statement

The datasets generated for this study can be found online at the NCBI Sequence Read Archive (SRA): https://www.ncbi.nlm.nih.gov/sra/PRJNA428620.

## Ethics Statement

Ethical review and approval was not required for the study on human participants in accordance with the local legislation and institutional requirements. Written informed consent for participation was not required for this study in accordance with the national legislation and the institutional requirements.

## Author Contributions

LG, JH, and CL wrote this manuscript and conceived the project study design. LG and JH selected the datasets. LG analyzed the sequence data and generated figures and tables. JH prepared DNA and sequenced the strains in this study. All authors contributed to the article and approved the submitted version.

### Conflict of Interest

The authors declare that the research was conducted in the absence of any commercial or financial relationships that could be construed as a potential conflict of interest.

## Footnotes

^1^https://www.cdc.gov/botulism/surveillance.html
