# Supplementary material for: Genomic Characterization of Strains From a Cluster of Infant Botulism Type A in a Small Town in Colorado, United States
Source: Front Microbiol. 2021 Jul 13;12:688240. doi: 10.3389/fmicb.2021.688240 (PMC8313963; doi:10.3389/fmicb.2021.688240)
Supplement: Supplementary file 1 [file Table_1.DOCX]

**SUPPLEMENTAL TABLE.** Unpublished *C. botulinum* strains not associated with the study strains and published NCBI references that that were included in this study.

| **Strain No./NCBI Accession (if applicable)** | **Botulism Type** | **Location** | **Toxin Subtype** |
| --- | --- | --- | --- |
| CDC21519 | Foodborne | AK | A1, B5 |
| CDC21520 | Foodborne | AK | A1, B5 |
| CDC32337T1 | Infant | DC | A1, B5 |
| CDC32337PL2 | Infant | DC | A1, B5 |
| CDC43305 | Foodborne | CT | A1, B5 |
| CDC43306 | Foodborne | CT | A1, B5 |
| CDC60006 | Infant | LA | A4, B5 |
| CDC60099B1 | Infant | NC | A1, B5 |
| CDC60099PL1 | Infant | NC | A1, B5 |
| CDC60101 | Unknown | CA | A1, B5 |
| CDC60256 | Infant | NJ | A1, B5 |
| CDC60283 | Infant | NJ | A1, B5 |
| CDC61017 | Infant | CO | A1, B5 |
| CDC61060 | Foodborne | OK | A1, B5 |
| CDC61111 | Foodborne | MS | A1, B5 |
| CDC61118H1 | Foodborne | MS | A1, B5 |
| CDC61118PL1 | Foodborne | MS | A1, B5 |
| CDC62001 | Infant | GA | A1, B5 |
| CDC63081 | Infant | NC | A1, B5 |
| CDC64138 | Infant | TX | A1, B5 |
| CDC64140 | Infant | TX | A1, B5 |
| CDC64144 | Infant | TX | A1, B5 |
| CDC64145 | Infant | TX | A1, B5 |
| CDC64216 | Foodborne | CO | A1, B5 |
| CDC64218 | Foodborne | CO | A1, B5 |
| CDC64223 | Foodborne | CO | A1, B5 |
| CDC64235 | Infant | IL | A1 |
| CDC69035 | Infant | TN | A1, B5 |
| CDC69080 | Infant | GA | A1, B5 |
| CDC75005 | Foodborne | TX | A1, B5 |
| CDC75008 | Infant | FL | A1, B5 |
| CDC75038 | Infant | SC | A1, B5 |
| CDC51348/ SAMN03769218 | Foodborne | FL | A1, B5 |
| CDC67190/ SAMN02664956 | Foodborne | AZ | A1, B5 |
| CDC69094/ SAMN04262333 | Infant | UT | A1, B5 |
| CJ0611A1/ SAMN13428652 | Foodborne | Canada | A1, B5 |
| 20391/SAMN03779957 | Foodborne | CO | A1, B5 |
| NCTC2916/SAMN02436237 | Foodborne | USA | A1, B5 |
